# Invertebrate Iridescent Virus 6, a DNA Virus, Stimulates a Mammalian Innate Immune Response through RIG-I-Like Receptors

**DOI:** 10.1371/journal.pone.0166088

**Published:** 2016-11-08

**Authors:** Laura R. H. Ahlers, Reginaldo G. Bastos, Aoi Hiroyasu, Alan G. Goodman

**Affiliations:** 1 School of Molecular Biosciences, College of Veterinary Medicine, Washington State University, Pullman, Washington, United States of America; 2 NIH Protein Biotechnology Graduate Training Program, Washington State University, Pullman, Washington, United States of America; University of Hong Kong, HONG KONG

## Abstract

Insects are not only major vectors of mammalian viruses, but are also host to insect-restricted viruses that can potentially be transmitted to mammals. While mammalian innate immune responses to arboviruses are well studied, less is known about how mammalian cells respond to viruses that are restricted to infect only invertebrates. Here we demonstrate that IIV-6, a DNA virus of the family *Iridoviridae*, is able to induce a type I interferon-dependent antiviral immune response in mammalian cells. Although IIV-6 is a DNA virus, we demonstrate that the immune response activated during IIV-6 infection is mediated by the RIG-I-like receptor (RLR) pathway, and not the canonical DNA sensing pathway via cGAS/STING. We further show that RNA polymerase III is required for maximal IFN-β secretion, suggesting that viral DNA is transcribed by this enzyme into an RNA species capable of activating the RLR pathway. Finally, we demonstrate that the RLR-driven mammalian innate immune response to IIV-6 is functionally capable of protecting cells from subsequent infection with the arboviruses Vesicular Stomatitis virus and Kunjin virus. These results represent a novel example of an invertebrate DNA virus activating a canonically RNA sensing pathway in the mammalian innate immune response, which reduces viral load of ensuing arboviral infection.

## Introduction

The innate immune response is composed of a set of defense mechanisms that protect the host from microbial pathogens. This initial sensing of pathogens occurs through the activation of host pattern recognition receptors (PRRs) by pathogen-associated molecular patterns (PAMPs), which are conserved molecular motifs unique to microbes or are generated during the pathogen’s cycle of infection [1]. Significant progress has been made during the past two decades in understanding how innate immune signaling pathways are activated and, in turn, shape adaptive immunity. Much of this research has focused on the immune response to viruses that are transmitted by an insect vector, such as West Nile virus (WNV) and Dengue virus (DENV). Despite these advances, it is currently unknown if insect-restricted viruses are able to also infect mammalian cells and initiate an immune response. This work is of importance because of the potential for emerging viruses to escape the immune response and adapt to new hosts. Furthermore, it is not well known how certain viruses, but not others, cross species and produce productive infections among diverse phyla.

The mammalian immune response has several mechanisms to sense viral infection by both DNA and RNA viruses through a variety of PRRs. The innate immune system responds to DNA viruses through cyclic GMP-AMP synthase (cGAS). cGAS is able to bind cytosolic DNA during virus infection, which triggers cGAS to metabolize ATP and GTP into non-canonical cyclic dinucleotides (CDNs). These non-canonical CDNs contain mixed 2’-5’ and 3’-5’ phosphodiester linkages which are then able to activate the adaptor protein stimulator of interferon genes (STING) [2–4]. The activation of STING leads to an interferon-mediated antiviral response [4–7] to viruses such as herpes simplex virus-1 (HSV-1) [6] and adenovirus [8]. Viral RNA sensing occurs primarily through the RIG-I-like receptors (RLRs) [9]. The family of RLRs contains three members involved in the immune response: Retinoic acid-inducible gene I (RIG-I) [10–11], melanoma differentiation-associated gene 5 (MDA5) [12–14], and laboratory of genetics and physiology 2 (LGP2) [15]. Of these three, RIG-I and MDA5 have been demonstrated to sense infection of RNA viruses and signal for an innate immune response, and LGP2 has been implicated in both positive and negative regulation of a RIG-I-mediated IFN-β response [11,16–18]. Although they are complementary and have some overlapping function, RIG-I and MDA5 respond to unique properties of viral RNA. RIG-I detects the 5’-triphosphate of single-stranded RNA [19–21] in conjunction with a short dsRNA region [22], and typically responds to sequences that are shorter in length [9]. RIG-I is implicated in the immune response to Hepatitis C virus [23], Vesicular Stomatitis virus (VSV) [19], and Japanese encephalitis virus [24]. MDA5 is known to detect longer RNA sequences that are double-stranded [9]. MDA5 is implicated in the immune response to encephalomyocarditis virus [19] and Sendai virus [25]. However, there is some overlap in viral recognition: Both RIG-I and MDA5 detect DENV [26] and WNV [27]. In this RNA sensing immune pathway, RIG-I or MDA5 senses viral nucleic acids [9], then signals through mitochondrial antiviral-signaling protein (MAVS) to activate the transcription factors interferon regulatory factor 3 (IRF3) and NFκB, leading to the induction of the cytokine IFN-β [28].

Host RNA polymerases also play a role in the activation of the innate immune response. RNA polymerase II is recruited by IRF3 and NFκB during Sendai virus infection to induce antiviral genes and achieve a protective immune response [29]. Furthermore, host RNA polymerase III (RNA Pol III) is a cytosolic DNA sensor that converts cytosolic AT-rich DNA into RNA to be sensed by RIG-I, leading to a MAVS-mediated IFN-β immune response [30]. RNA Pol III contributes to activation of the immune response to HSV-1 [30] and Epstein-Barr virus (EBV) [31]. Both the MAVS pathway and cGAS/STING pathway lead to the phosphorylation of IRF3, a transcription factor that induces IFN-β [32]. Secreted IFN-β acts as a second messenger to signal to interferon-stimulated genes (ISGs), such as the JAK-STAT pathway, to further activate the antiviral immune response or apoptotic pathways [33–34].

In this study, we use Invertebrate Iridescent Virus 6 (IIV-6) (also called Chilo Iridescent Virus and Insect Iridescent Virus 6) to probe an immune response in mammalian cells. Because this virus is known to infect and replicate in invertebrates, this work asks if evolutionarily conserved nucleic acid sensing pathways are activated in mammals in response to an invertebrate DNA virus. IIV-6 is a DNA virus of the *Iridoviridae* family [35], and has a large circular 212.5 kb dsDNA genome containing 234 open reading frames [36]. During IIV-6 infection in *Drosophila*, Dicer-2, whose RNA helicase domain is well conserved compared to RIG-I [37], has been shown to initiate powerful antiviral activity through the RNA interference (RNAi) pathway to restrict IIV-6 infection [38]. Since RIG-I is absent in invertebrates and Dicer acts as the antiviral RNA sensor, in this work we sought to address the converse and ask if the invertebrate virus IIV-6 activated an evolutionarily conserved RNA sensing pathway in mammalian cells. Specifically, we infected mammalian cells with IIV-6 in an attempt to identify potential innate immune mechanisms that could prevent IIV-6 from establishing a productive infection in mammals. In doing so, we revealed a novel example of a DNA insect virus eliciting an IFN-β immune response that occurs through the RIG-I pathway. We also show that host RNA Pol III activity is required for maximal activation of the IFN-β response. Additionally, by demonstrating that the mammalian immune response to IIV-6 restricts infection by the viruses VSV and Kunjin virus (KUNV), a subtype of WNV, we show that IIV-6-mediated activation of the mammalian innate immune response reduces viral load during subsequent arbovirus infection.

## Materials and Methods

### Ethics statement

All animal work was conducted in accordance with the Guide for the Care and Use of Laboratory Animals, the American Veterinary Medical Association (AVMA), and approved by the Institutional Animal Care and Use Committee (IACUC) at Washington State University, which is fully accredited by the American Association for Accreditation of Laboratory Animal Care.

### Cell lines

Primary wild-type (WT) mouse embryonic fibroblasts (MEFs) were collected from d13.5 embryos of C57BL6 mice. Pregnant female mice at 13.5 d post-coitus were euthanized with isoflurane followed by cervical dislocation. Uterine horns containing embryos were dissected out and placed in PBS. Embryos were then removed from the uterine sac, and the placentas, red organs, and heads were removed. Remaining embryonic tissue was washed in PBS and minced with a razor blade. Tissue was placed in 1 mL trypsin-EDTA (0.05%) (ThermoFisher 25300054) per embryo and incubated at 37°C for 15 min with vigorous pipetting every 5 min. Following incubation, 1 volume of cell culture media (see below) was added and cells were centrifuged at 300 RCF for 5 min. Cells were resuspended in cell culture media and fatty/collagenous tissue was removed by passing resuspension through a 100 μm filter. 3–4 embryo equivalents were added to a T150 flask precoated with 0.1% rat tail collagen I (Corning 354236). 24 hours later, cells were removed by trypsin and aliquots were frozen in cell culture media containing 10% DMSO and stored in liquid nitrogen for future use.

Primary STING^-/-^ MEFs from C57BL6 mice were kindly provided by D. Stetson [39]. MAVS^+/+^, MAVS^-/-^ [40], RIG-I^+/+^, RIG-I^-/-^ [41], MDA5^+/+^ and MDA5^-/-^ [42] MEFs were kindly provided by S. Balachandran. Dicer^-/-^ MEFs were kindly provided by M. Otsuka [43]. MEFs, HEK 293T cells, BHK21 cells, human lung A549 cells, and RAW 264.7 macrophages were cultured in DMEM (ThermoFisher 11965118) with 10% FBS (ThermoFisher SH3007003HI) and antibiotic-antimycotic (ThermoFisher 15240062) at 37°C and 5% CO_2_. S2 cells were cultured in Schneider’s Drosophila Medium (ThermoFisher 21720024) with 10% FBS and antibiotic-antimycotic at 28°C. S2 cells are negative for the presence of Flock House virus, determined by qRT-PCR using primers for the gene coding for B2. Fwd: CAAGCAAACTCGCGCTAATC; Rev: GCGTCTTGGTAGCTCATTCC.

### Viruses

IIV-6 and DCV kindly provided by L. Teixeira were grown in S2 cells and purified by ultracentrifugation, as previously described [44–45]. A mock purification of IIV-6 (PBS inoculation of S2 cells followed by same protocol to purify IIV-6) was used to test for contaminants from the viral purification protocol that could elicit an immune response. MEFs infected with mock IIV-6 purification did not elicit an IFN-β immune response, as measured by ELISA (data not shown). IIV-6 was titrated on S2 cells to determine viral titer by end-point dilution [46]. Heat-inactivated IIV-6 was prepared by heating the virus at 80°C for 30 minutes. UV-inactivation was achieved by treating virions with 10 cycles of 1 J/cm^2^ of UV-C light [47]. VSV (Indiana Strain) and KUNV (strain MRM16, provided by R. Tesh) were propagated and titrated on BHK21 cells by standard plaque assay. Insect virus infections were performed in cells seeded onto plates at 37°C/5% CO_2_, washed once with PBS, then infected with virus at an MOI of 1 TCID_50_/cell for one hour at 28°C. The cells were washed three times with PBS, and the cell media was replaced with 2% FBS/DMEM. Cells remained at 37°C/5% CO_2_ for the remainder of the experiments. For the IIV-6 priming experiments, supernatant from IIV-6-infected MEFs was first centrifuged at 15,000 RCF for 10 minutes to pellet virus present in the cell culture supernatant [44].

### Analysis of immune response

Supernatant from infected cells was collected, and ELISA was performed using a mouse IFN-β serum kit (PBL Assay Science 42410–1) following manufacturer’s instructions. ISRE-reporter assay for A549 cells was performed by transfecting cells with 1 μg/mL of poly(dA:dT) or infected with IIV-6 at an MOI of 1 TCID_50_/mL in biological triplicate. At the indicated times, cell culture supernatant was collected. HEK 293T cells were transfected with 80 ng/mL of ISRE-firefly luciferase and 80 ng/mL of TK-Renilla luciferase. 6 hours post-transfection, the cell culture media was replaced with cell culture supernatant collected from infected A549 cells. After 16 hours of incubation, cells were lysed and firefly luminescence was measured and normalized to TK-Renilla.

### Quantitative reverse transcriptase PCR

qRT-PCR was used to measure cytokine mRNA levels with primer-probe sets for IFN-β (*Ifnb1*) (ThermoFisher Mm00439552_s1) and TNF-α (*Tnf*) (ThermoFisher Mm00443258_m1), as well as to confirm gene knockdown of RNA Pol III (*Polr3d*) (ThermoFisher Mm00508948_g1), RIG-I (*Ddx58*) (ThermoFisher Mm01216853_m1), and MDA5 (*Ifih1*) (ThermoFisher Mm00459183_m1), using TaqMan Universal PCR Master Mix (ThermoFisher 4304437). Infected cells were lysed with Solution D (4 M guanidinium thiocyanate, 25 mM sodium citrate, 0.5% sarcosyl, 0.7% β-mercaptoethanol). RNA was purified from cell lysates and cDNA was prepared (ThermoFisher K0732 and 18068, and BioRad 170–8891). qRT-PCR was performed for gene expression, using mouse β-actin (ThermoFisher Mm00607939_s1) for normalization and ROX (ThermoFisher 12223–012) as an internal control. The qRT-PCR reaction initialized at 95°C for 10 minutes. The reaction then cycled 40 times between denaturation at 95°C for 15 seconds and extension at 60°C for 1 minute.

qPCR was used to measure copies of the IIV-6 genome in MEFs and S2 cells. A standard curve for absolute quantification was made by cloning the gene for IIV-6 capsid into a pCR’4-TOPO-TA vector. 24-well plates were seeded with 1x10^5^ MEFs or 2x10^5^ S2 cells and infected with IIV-6 at an MOI of 1 TCID_50_/cell. At various times post-infection, the cells were collected in PBS by scraping them off of the well plate. Genomic DNA was purified using the QIAGEN QIAmp DNA Mini Kit (QIAGEN 51304) following manufacturer instructions. qPCR was used to measure IIV-6 capsid levels using SYBR green (ThermoFisher K0251) with ROX as an internal control. The following primers were used to measure IIV-6 capsid Fwd: TACAACACCTGCGTCAAAGG; Rev: TGCAGGAGCAACAGGTACAG. Absolute levels of IIV-6 capsid were determined using the standard curve. The qPCR reaction initialized at 95°C for 10 minutes. The reaction then cycled 40 times between denaturation at 95°C for 15 seconds and extension at 60°C for 1 minute. Efficiency of amplification and melt curve analyses were performed to evaluate analytical sensitivity and specificity of the qPCR primers.

### Immunoblotting

Protein extracts were prepared by lysing cells with RIPA buffer (25 mM Tris-HCl (pH 7.6), 150 mM NaCl, 1 mM EDTA, 1% NP-40, 1% sodium deoxycholate, 0.1% SDS, 1 mM Na_3_VO_4_, 1 mM NaF, 0.1 mM PMSF, 10 μM aprotinin, 5 μg/mL leupeptin, 1 μg/mL pepstatin A). Protein samples were diluted using 2x Laemmli loading buffer, mixed, and boiled for 5 minutes at 95°C. Samples were analyzed by SDS/PAGE using a 10% acrylamide gel, followed by transfer onto PVDF membranes (Millipore IPVH00010). Membranes were blocked with 5% BSA (ThermoFisher) in Tris-buffered saline (50 mM Tris-HCl pH 7.5, 150 mM NaCl) and 0.1% Tween-20 for 1 hour at room temperature. Primary antibody labeling was done with anti-IRF3 (1:1,000) (Cell Signaling 4302S), anti-P-IRF3 (1:2,000) (Cell Signaling 4947S), anti-STAT1 (1:1,000) (EMD Millipore 06–501), anti-P-STAT (1:1,000) (Cell Signaling 7649), anti-IκB (1:1,000) (Santa Cruz sc-371), anti-P-IκB (1:1,000) (Cell Signaling 2859), anti-actin (1:10,000) (Sigma A2066), or anti-human IRF3 (1:500) [48] overnight at 4°C. Secondary antibody labeling was done using anti-rabbit or anti-mouse IgG-HRP conjugate (1:10,000) (Promega W4018, W4021) by incubating membranes for 2 hours at room temperature. Blots were imaged onto film using luminol enhancer (ThermoFisher 1859675).

### Microscopy

Samples for confocal microscopy were prepared by seeding cells onto coverslips, infecting with IIV-6 at an MOI of 1 TCID_50_/cell, and fixing at 8 hours post-infection with 4% paraformaldehyde for 20 minutes. Coverslips were washed in PBS, permeabilized with 0.1% Triton-X, and blocked with 10% FBS in PBS. Cells were labeled for NFκB nuclear translocation by incubating in primary NFκB antibody (1:50) (Santa Cruz sc-372) for one hour, followed by incubating in secondary antibody conjugated to Alexa Fluor 488 (1:200) (ThermoFisher A11034) for one hour. Washed cells were then incubated in DAPI for one hour, and then mounted onto microscope slides (ThermoFisher P36961). Slides were imaged using a Leica SP5 confocal microscope.

Samples for transmission electron microscopy (TEM) were prepared by seeding cells onto plates and infecting with IIV-6 at an MOI of 1 TCID_50_/cell. Cells were fixed in 2% paraformaldehyde/2% glutaraldehyde in 0.1 M cacodylate buffer with 0.2 M sucrose, dehydrated in ethanol, infiltrated with Spurrs’ resin, sectioned, and stained with 4% uranyl acetate and Reynolds lead. Imaging was done at the Franceschi Microscopy and Imaging Center on a FEI Tecnai G2 20 Twin microscope.

Cells for live-cell imaging with phase contrast microscopy were seeded and infected with IIV-6 at an MOI of 1 TCID_50_/cell. Infected MEFs were visualized at 24 hours post-infection with a Leica DMi8 phase contrast/DIC microscope. Brightfield imaging was performed with a Cytation 3 Multi-Mode Reader (Biotek).

### RNA interference and RNA Pol III inhibition

For siRNA knockdown experiments, MEFs were seeded in 24-well plates and transfected with 0.15 μM siRNA and Lipofectamine RNAimax (Life Technologies 13778030) for 72 hours using the following siRNA constructs for mouse cells: RNA Pol III (*Polr3d*) (Dharmacon L-063472-01-0005), RIG-I (*Ddx58*) (Dharmacon L-065328-00-0005), MDA5 (*Ifih1*) (Dharmacon L-048303-00-0005) or non-targeting control (Dharmacon D-001810-10-05). RNA Pol III was inhibited by treating cells with 100 μM ML-60218 (EMD Millipore 557403) for 12 hours [49].

### Viral DNA isolation and transfection

Genomic DNA was purified from 2.7x10^6^ TCID_50_ IIV-6 using the QIAamp DNA mini kit including RNase A treatment. MEFs were seeded at 1x10^5^ cells in a 24-well plate and transfected with 1 μg DNA using Lipofectamine 2000 (ThermoFisher 11668027) following manufacturer instructions.

### Quantification of cell death

1x10^5^ MEFs were seeded in a 24-well plate and infected with IIV-6 for 24 h. Cells were trypsinized and the well was washed with PBS to collect all cells. All trypsinized cells and washes were collected into the same tube, centrifuged, and resuspended in 0.04% trypan blue (ThermoFisher 15250–061) in PBS. Total and dead cells were counted using a hemocytometer, with the dead cells being distinguishable by membranes stained blue. Similarly, MEFs were infected, collected, and resuspended for flow cytometry. Propidium iodide (final concentration 0.2 μg/mL) (ThermoFisher V3241) was used to quantify cell death using a Guava easyCyte flow cytometer.

### Statistical analysis

Statistical analysis was performed by two-tailed Student’s T-test assuming unequal variance (GraphPad Prism 6). Graphed bars or data points represent the mean and the error bars represent standard deviation of the mean.

## Results

### IIV-6 infection elicits an immune response in mammalian cells

**Fig 1:**
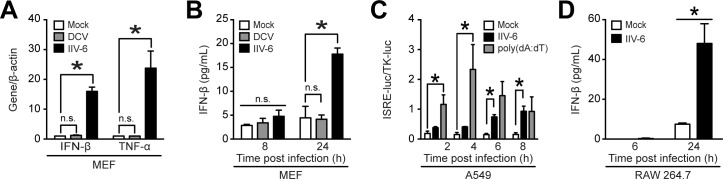
IIV-6 elicits a type I IFN response in mammalian cells. (A-B) MEFs, (C) human A549 cells, or (D) mouse RAW 264.7 macrophages were infected with IIV-6 or DCV at an MOI of 1 TCID_50_/cell. 24 hours post-infection supernatant and total RNA were collected to measure (A) IFN-β and TNF-α induction by qRT-PCR, (B, D) IFN-β secretion by ELISA, or (C) ISRE-luciferase activity in HEK 293T cells. Poly(dA:dT) was transfected into A549 cells with lipofectamine as a positive control for ISRE activity. (D) Mock-infected cells did not secrete significantly higher levels of IFN-β at 24 hours as compared to either mock- or IIV-6-infected cells at 6 hours. ELISA and qRT-PCR assays were performed in biological duplicate, and ISRE-luciferase assay for IFN-β secretion from A549 cells was completed in biological triplicate (*, *P* < 0.05).

Iridoviruses, such as IIV-6, and Dicistroviruses, such as *Drosophila* C virus (DCV), infect insects and cold-blooded vertebrates [50–52]. However, it is currently unknown if they are able to infect and initiate an immune response in mammalian cells similarly to arthropod-borne viruses, for example WNV and DENV. In this study, we investigated the ability of IIV-6 or DCV to infect and activate the mammalian immune response. As secretion of type I interferon (IFN) is a classical indication of immune activation upon viral infection [53], we first infected mouse embryonic fibroblasts (MEFs) with IIV-6 or DCV and investigated the induction of IFN-β and tumor necrosis factor α (TNF-α) as markers for an early innate immune antiviral response in infected cells. We observed a 15-fold increase in IFN-β mRNA and a 23-fold increase in TNF-α mRNA in IIV-6-infected cells compared to mock-infected cells 24 hours post-infection (Fig 1A). Contrarily, we did not observe significant induction of IFN-β and TNF-α in MEFs infected with DCV (Fig 1A). We also examined secreted IFN-β from the cell culture supernatant as an indication an innate immune response, and observed that IFN-β is secreted in response to IIV-6, but not DCV in infected MEFs (Fig 1B). Additionally, we observed significant reduction of IFN-β secretion in MEFs exposed to heat- or UV-inactivated IIV-6 (S1 Fig), indicating that non-denatured viral proteins and genomic nucleic acids are necessary to elicit IFN-β secretion in MEFs. These methods of denaturation could inhibit the IFN-β response due to loss of viral entry, viral replication, or viral DNA transcription. Next, we infected human A549 lung epithelial cells with IIV-6 and used cell culture supernatant to measure the induction of the human IFN-stimulated response element (ISRE) with human embryonic kidney (HEK) 293T cells [54]. By 6 hours post-infection, supernatant from IIV-6-infected A549 cells significantly activated an ISRE-driven reporter construct (Fig 1C). We transfected poly(dA:dT) into A549 cells as a positive control for ISRE activity. Additionally, RAW 264.7 mouse macrophages secreted significant levels of IFN-β when infected with IIV-6, as detected by ELISA (Fig 1D). Mock-infected RAW 264.7 cells secreted low levels of IFN-β at 24 hours post-infection, and we hypothesize that it is due to the cells being cultured in media supplemented with only 2% FBS or because the cells are reaching confluency, each of which may initiate a stress-induced immune response.

**Fig 2:**
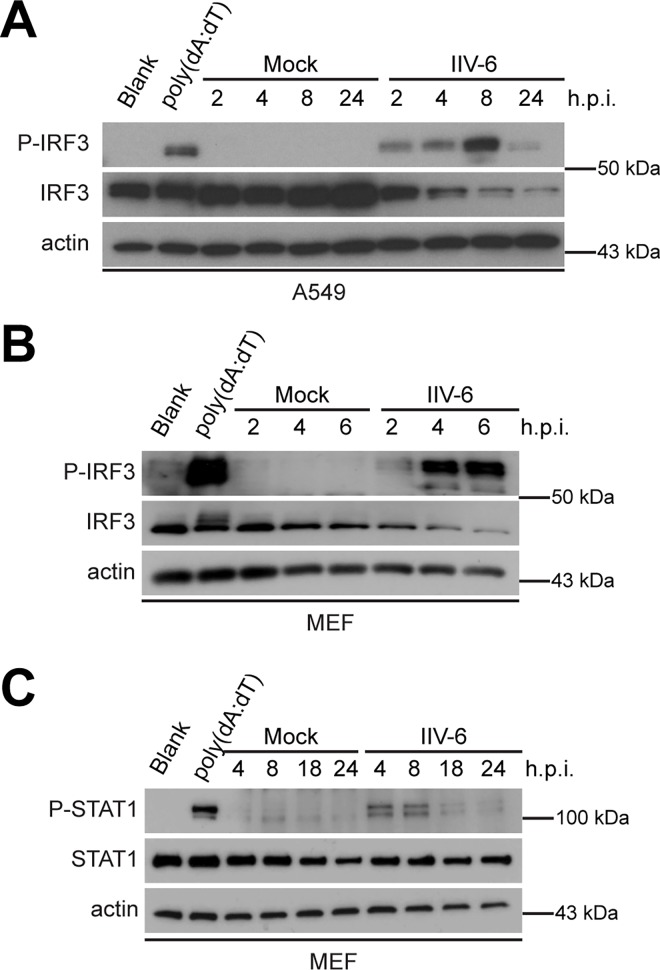
IRF3 and STAT1 are activated during IIV-6 infection in mammalian cells. (A) A549 cells or (B-C) MEFs were infected with IIV-6 at an MOI of 1 TCID_50_/cell. Cellular protein lysates were analyzed by Western blot for phosphorylated IRF3 and STAT1, total IRF3 and STAT1, and actin. 1 μg/mL poly(dA:dT) was transfected into cells using lipofectamine as a positive control, and blank lipofectamine was used as a negative control.

**Fig 3:**
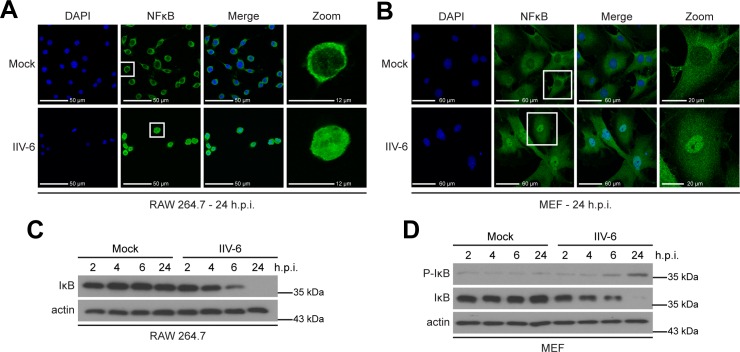
NFκB is activated during IIV-6 infection. (A, C) RAW 264.7 macrophages or (B, D) MEFs were infected with IIV-6 at an MOI of 10 TCID_50_/cell. (A-B) 24 hours post-infection cells were fixed onto coverslips, and stained for NFκB and DAPI. (C-D) Protein samples were analyzed using Western blot for phosphorylated IκB, total IκB, and actin.

We next investigated the transcription factors involved in the induction of IFN-β and asked if IRF3 was phosphorylated in IIV-6-infected cells, indicating the activation of this transcription factor during infection in both MEFs and A549 cells. We determined that by 8 hours post-infection in A549 cells and 6 hours post-infection in MEFs, IRF3 was maximally phosphorylated in response to IIV-6 infection (Fig 2A and 2B). We also observed the reduction of total IRF3 as the protein is degraded by the proteasome following activation [55]. Following IRF3 activation and IFN-β induction, IFN-β engages the type I IFN receptor and activates the JAK-STAT pathway [56]. Therefore, we additionally determined that STAT1 is phosphorylated and activated in MEFs following IIV-6 infection (Fig 2C). As NFκB activation also contributes to the interferon-mediated immune response via a positive feedback loop [57–59], we observed nuclear translocation of NFκB in response to IIV-6 infection in both RAW 264.7 macrophages and MEFs, as depicted by its overlap with the nucleus (Fig 3A and 3B). We also observe degradation of the Inhibitor of κB (IκB) [60–61] following IIV-6 infection (Fig 3C and 3D) by 24 hours post-infection, the time at which NFκB nuclear translocation occurs.

**Fig 4:**
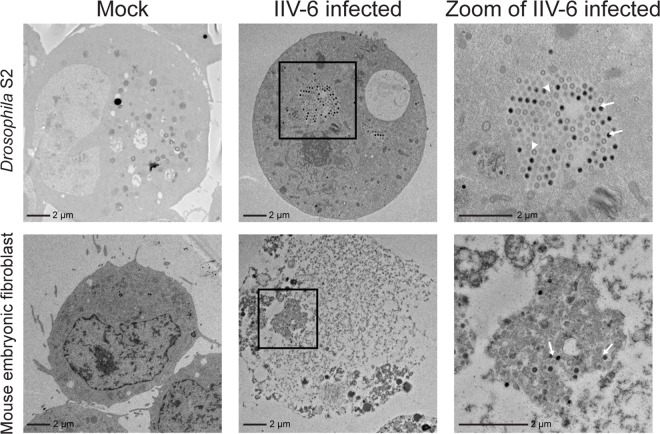
IIV-6 enters mouse embryonic fibroblasts. S2 cells and MEFs were infected with IIV-6 at an MOI of 1 TCID_50_/cell. S2 cells were fixed at 24 hours post-infection and MEFs were fixed at 72 hours post-infection. The top row indicates S2 controls for the presence of IIV-6, and the zoom of infected S2 cells illustrates the presence of a viral factory in which capsids are developing. Arrows indicate representative virions, and arrowheads indicate empty capsids. The bottom row depicts MEFs, and the zoom indicates the presence of viral particles.

The presence of viral factories in the cytoplasm of infected cells is a classical indication of productive Iridovirus infection [62]. After demonstrating activation of the immune response by IIV-6 infection, we performed transmission electron microscopy (TEM) to investigate the presence of viral particles inside mammalian cells. We observed IIV-6 particles in a low percentage of MEFs at 72 hours post-infection, indicating viral entry (Fig 4, bottom row). The IIV-6 particles in S2 cells were organized in typical viral factories showing the expected pattern of Iridovirus morphogenesis including complete, developing, and empty particles [62] (Fig 4 top row). IIV-6 particles in infected MEFs did not arrange into factory structures (Fig 4, bottom row). However, MEFs showed cytopathic effects (CPE) 24 hours post-infection as demonstrated by phase contrast microscopy, trypan blue exclusion, and flow cytometry (S2 Fig).

Additionally, we performed qPCR to quantify IIV-6 genome levels in MEFs. We observed no increase in IIV-6 genome copies during a 6-day period (S3A Fig). Nevertheless, the viral genome increased significantly from the time of infection in S2 cells during the same period of infection (S3B Fig). Supernatant or cell lysate from MEFs infected with IIV-6 titered onto S2 cells did not reveal an increase in virus TCID_50_, confirming the absence of IIV-6 replication in MEFs (S3C and S3D Fig, respectively). Collectively these results indicate that IIV-6 entry elicits an immune response in mammalian cells but that there is an absence of productive viral replication.

### RIG-I mediates the immune response to IIV-6 in MEFs

**Fig 5:**
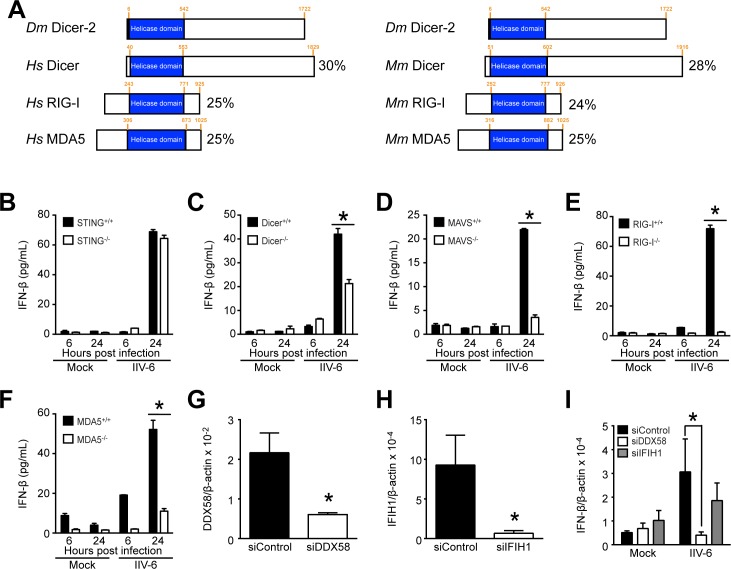
IFN-β secretion following IIV-6 infection is reduced in the absence of RLRs and Dicer. (A) Schematic representing the homologous DExD/H-box helicase domains shared by *Drosophila* Dicer-2, and human or mouse Dicer, RIG-I, and MDA5. Orange numbers indicate the amino acid residues where the helicase domain begins and ends and the total length of the protein. Percentages on the right are the percent identity of the amino acid sequence of *Drosophila* Dicer-2 to the mammalian protein in the helicase domain determined by Clustal Omega alignment. (B-F) STING^-/-^, Dicer^-/-^, MAVS^-/-^, RIG-I^-/-^, or MDA5^-/-^ MEFs and their corresponding wild-type MEFs were infected with IIV-6 at an MOI of 1 TCID_50_/cell in biological duplicate. Supernatant from cells was collected at 6 and 24 hours post-infection and analyzed for secreted IFN-β by ELISA. (G-H) *Ddx58* and *Ifih1*, the genes encoding RIG-I and MDA5, respectively, were knocked down in MEFs using siRNAs, as determined by qRT-PCR. (I) 72 hours after siRNA transfection, MEFs were infected with IIV-6 at an MOI of 1 TCID_50_/cell. Total RNA was collected for qRT-PCR 24 hours post-infection to measure IFN-β gene induction in biological triplicate. (B-F) Knockout cell lines were compared to the wild-type parental line at each time point, and (I) IFN-β expression for siDDX58 or siIFIH1 was compared to siControl (*, *P* < 0.05).

Considering that IIV-6 is a DNA virus, we first hypothesized that the mammalian immune response to IIV-6 would occur through the cGAS/STING pathway, which is the canonical DNA-sensing pathway. However, we observed no change in IFN-β levels between STING^-/-^ MEFs and wild-type MEFs, as measured by ELISA for IFN-β secretion (Fig 5B), or qRT-PCR for cytokine mRNA expression (S4A Fig), indicating that the immune response to IIV-6 is independent of the STING pathway.

It has previously been demonstrated that Dicer-2 in *Drosophila* has a DExD/H-box helicase domain that is homologous to the RLRs RIG-I and MDA5 [37]. Dicer-2 has been shown to initiate powerful antiviral activity through the RNA interference (RNAi) pathway [38,63]. Dicer-2 detects viral RNA during infections with WNV [64], DENV [65], DCV [66–67], and VSV [68], and it also responds to the DNA virus IIV-6 [38]. Taken together, nucleic acid sensing and the activation of the RNAi pathway is a major antiviral defense mechanism in insects. As previously determined by Deddouche et al, comparison of amino acid sequences using the Clustal Omega alignment tool, reveals that human or mouse Dicer, RIG-I, and MDA5 have 24–30% identity with *Drosophila* Dicer-2 in the helicase domain, indicating sequence conservation among the RNA-binding domains of these proteins (Fig 5A) [37]. Given this demonstrated domain similarity and the dependence of the fruit fly on Dicer-2 for an immune response to IIV-6 infection, we asked if the related proteins RIG-I, MDA5, and mammalian Dicer function in the immune response to IIV-6. We infected MEFs from RIG-I, MDA5, Dicer, or MAVS knockout mice with IIV-6 and quantified secreted IFN-β by ELISA. Levels of secreted IFN-β were significantly reduced in all infected knockout cell lines compared to parental wild-type MEFs by 24 hours post-infection (Fig 5C–5F). To corroborate the ELISA data, we collected RNA from infected MEFs at 24 hours post-infection and analyzed the samples by qRT-PCR for IFN-β induction. The expression of IFN-β mRNA was significantly higher in wild-type MEFs as compared to RIG-I^-/-^ MEFs. However, IFN-β mRNA levels were not significantly different in MDA5^-/-^, Dicer^-/-^, or MAVS^-/-^ MEFs compared to parent lines (S4B–S4E Fig). The lack of significantly reduced IFN-β induction in MDA5^-/-^, Dicer^-/-^, or MAVS^-/-^ MEFs led us to hypothesize that MDA5, MAVS, and Dicer play less of a role than RIG-I in modulating the immune response to IIV-6. We further examined the roles of RIG-I and MDA5 through RNAi knockdown experiments in MEFs (Fig 5G–5I) followed by IIV-6 infection. While we observed significant down-regulation of IFN-β induction when RIG-I was knocked down, knockdown of MDA5 did not significantly reduce IFN-β levels compared to control RNAi, as determined by qRT-PCR (Fig 5I). Based on the result that RIG-I significantly contributes to an innate immune response in IIV-6-infected MEFs, we asked if IIV-6 would replicate in RIG-I^-/-^ MEFs; however, like in wild-type MEFs, we observed no significant increase in IIV-6 replication in RIG-I deficient MEFs (S5 Fig). Taken together, these results indicate that IFN-β induction is mediated by RIG-I during IIV-6 infection and that MDA5, MAVS, and Dicer likely play a lesser role, in the activation of an IFN-β response during IIV-6 infection. However, the loss of RIG-I was not sufficient to rescue IIV-6 replication in mammalian cells, indicating that the lack of replication is due to factors other than the mammalian innate immune response.

### RNA polymerase III is required for maximal IFN-β secretion in MEFs following IIV-6 infection

**Fig 6:**
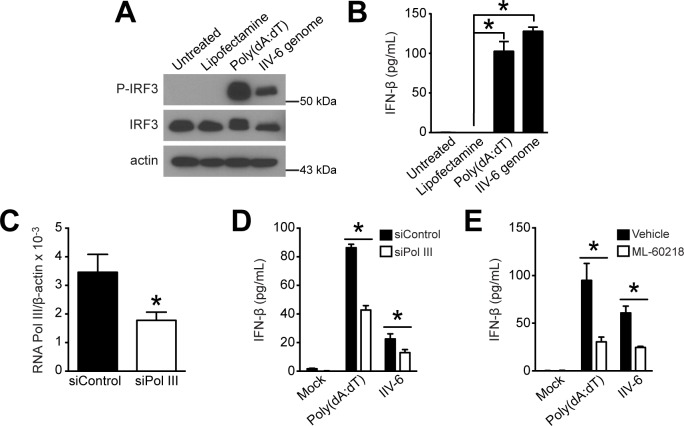
Host RNA polymerase III activity contributes to IFN-β activation following IIV-6 infection. (A-B) Genomic DNA was extracted from purified IIV-6 particles, treated with RNase A, and transfected into MEFs at 1 μg/mL in biological triplicate. (A) Protein was collected at 6 hours post-infection, triplicate protein samples were combined, and samples were analyzed by Western blot for IRF3 activation. Poly(dA:dT) was transfected as a positive control for IRF3 activation. (B) Supernatant was collected at 24 hours post-infection and analyzed by ELISA for secreted IFN-β. (C-D) MEFs were transfected with control siRNA (siControl) or siRNA targeting RNA Pol III (siPol III) for 72 hours and (C) total RNA was collected to measure RNA Pol III levels by qRT-PCR. (D) After RNA Pol III knockdown, cells were infected with IIV-6 at an MOI of 1 TCID_50_/cell. IFN-β secretion was measured by ELISA 24 hours post-infection. (E) RNA Pol III in MEFs was inhibited by treating cells with 100 μM ML-60218 for 12 hours prior to infection with IIV-6 or transfection with poly(dA:dT). Samples for ELISA and qRT-PCR were measured in biological triplicate. IFN-β induction was compared to (B) blank lipofectamine or (D) siControl (*, *P* < 0.05).

Because IIV-6 has a DNA genome, we sought to confirm that viral DNA is indeed contributing to the activation of the mammalian immune response. We isolated genomic DNA from IIV-6, treated it with RNase A, and transfected it into MEFs. We collected protein at 6 hours post-transfection and analyzed samples for IRF3 activation by Western blot (Fig 6A). We also collected supernatant at 24 hours post-transfection and analyzed samples for IFN-β secretion with ELISA (Fig 6B). Collectively, these results indicate that viral genomic DNA stimulates an immune response in MEFs by activating IRF3 in MEFs, leading to IFN-β secretion.

Our findings suggest that the immune response to IIV-6 utilizes the RNA sensor RIG-I to elicit an innate immune response in MEFs. However, since IIV-6 is a DNA virus, there was a disconnect in our model of the innate immune response between viral entry and RIG-I sensing. RNA Pol III is a cytosolic DNA sensor that transcribes cytosolic AT-rich DNA from viruses into RNA that can act as a PAMP for a RIG-I-mediated immune response [30–31]. To determine if the DNA virus IIV-6 induces IFN-β through an RNA-sensing pathway via RNA Pol III-dependent transcription, we used siRNA knockdown of RNA Pol III to investigate its role in IFN-β induction following IIV-6 infection. We demonstrated that silencing RNA Pol III by RNA interference (Fig 6C) significantly decreased the production of secreted IFN-β in MEFs during IIV-6 infection and poly(dA:dT) transfection (Fig 6D). We corroborated these findings using ML-60218, an inhibitor of RNA Pol III [49]. We determined that IFN-β secretion is reduced during IIV-6 infection and poly(dA:dT) transfection when RNA Pol III is inhibited (Fig 6E). These results suggest that RNA Pol III acts as a viral DNA sensor during IIV-6 infection in MEFs and is utilized for the transcription of IIV-6 DNA to RNA for a RIG-I-mediated immune response.

### IIV-6 reduces viral load of secondary arbovirus infection in MEFs

**Fig 7:**
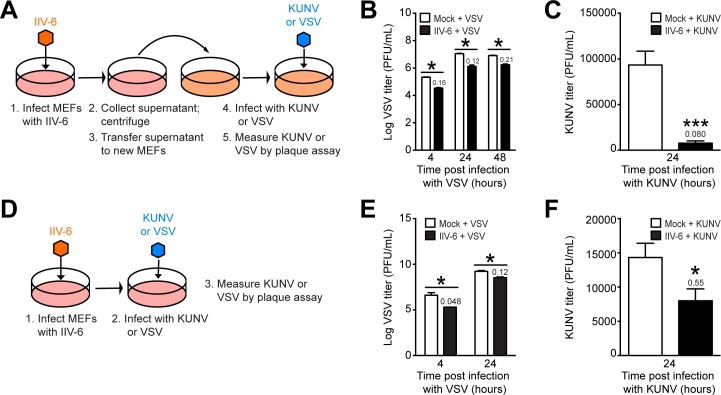
The mammalian immune response to IIV-6 restricts arbovirus infection. (A) Schematic of priming experiment: MEFs were infected with IIV-6 at an MOI of 1 TCID_50_/cell. 24 hours post-infection cell culture supernatant was removed, and viral particles in the cell culture supernatant were removed by centrifugation. Supernatant was used to prime new MEFs for 24 hours. Following priming, MEFs were infected with VSV or KUNV at an MOI of 1 PFU/cell, and cell culture supernatant was collected. (B-C) At the indicated time points post-infection, supernatant was collected for plaque assay on BHK21 cells to determine titers of (B) VSV or (C) KUNV. (D) Schematic of co-infection: MEFs were infected with IIV-6 at an MOI of 1 TCID_50_/cell for 1 hour, followed by infection with VSV or KUNV at an MOI of 1 PFU/cell, and cell culture supernatant was collected following infection for plaque assay. (E-F) At various times, supernatant was collected for plaque assay on BHK21 cells to measure titer of (E) VSV or (F) KUNV. Values above black bars (B-C, E-F) represent the fold-change decrease as compared to time-matched mock-primed MEFs. Assays with VSV were completed in biological duplicate, and assays with KUNV were completed in biological triplicate. Mock infection was compared to IIV-6 infection or priming for each time point (* *P* < 0.05; *** *P* < 0.001).

We next investigated the effect of IIV-6 infection on the susceptibility and permissiveness of MEFs to the arboviruses Vesicular Stomatitis virus (VSV) and Kunjin virus (KUNV). Two approaches were utilized to evaluate the effect of the antiviral response on subsequent arbovirus infection. In the first approach, we collected supernatant from IIV-6-infected MEFs 24 hours post-infection, removed IIV-6 particles by centrifugation (S6A Fig), and used the supernatant to prime fresh MEFs. After 24 hours of priming, we infected cells with either VSV or KUNV, and collected supernatants at different time points post-infection for VSV or KUNV titration (Fig 7A). Results demonstrated that MEFs primed with IIV-6-infected supernatants were significantly more resistant to VSV (Fig 7B) or KUNV (Fig 7C) infection compared to controls, with fold-change decreases up to eight-fold for VSV and twelve-fold for KUNV. Cell viability analysis indicates a significant reduction in VSV-induced cell death when MEFs were first primed with supernatant from IIV-6 infected cells (S6B Fig). In the second approach, we co-infected MEFs with IIV-6 and either VSV or KUNV, and collected supernatants at different time points for VSV or KUNV titration (Fig 7D). MEFs infected with IIV-6 were more resistant to co-infection with VSV (Fig 7E) or KUNV (Fig 7F) than control cells, with fold-change decreases up to twenty-fold for VSV and almost two-fold for KUNV. However, cell viability remained unchanged following co-infection with either VSV or KUNV (S6C Fig). Altogether, these data indicate that IIV-6 infection reduces virus replication in MEFs making the cells significantly more resistant to VSV or KUNV infection. This could be due to reduced VSV or KUNV entry following IIV-6 infection or due to the immunostimulatory properties induced by IIV-6. Taken together, IIV-6 could be used in the development of a method to restrict arbovirus infection in mammals since IIV-6 itself does not replicate in mammalian hosts.

## Discussion

In this study, we used IIV-6 to determine if insect viruses activate innate immune responses in mammalian cells. Viruses endemic to one species may fail to replicate in a different host species due to a lack of a receptor for virus entry [69], lack of compatibility with host machinery for genome replication [70], or by activation of the innate immune response [71]. These aspects, among others, are reasons for host restriction of viruses and the inability of a virus to replicate in other host species. Nevertheless, a question remains whether a virus that fails to infect a different host species is still able to activate an immune response. We show that IIV-6, but not the insect virus DCV, elicits a type I IFN response in mammalian cells; surprisingly, this response is mediated by the RLR pathway and not via cGAS/STING. Mechanistically, we find that the IFN-β-mediated immune response is due, in part, to host RNA Pol III activity that transcribes immunostimulatory RNA from viral DNA, which follows from the fact that the IIV-6 genome is 71% AT-content [72]. This is a novel example of an insect DNA virus activating an RNA-sensing pathway in mammalian cells. We further demonstrate the antiviral immune response to IIV-6 in mammals can reduce viral replication of the arboviruses VSV and KUNV.

Our data led us to develop a working hypothesis of how IIV-6 initiates the innate immune response in mammalian cells. In this model, RNA Pol III transcribes AT-rich viral DNA into RNA, which is then sensed by RIG-I, likely due to the presence of the uncapped 5’ppp, leading to an IFN-β-mediated immune response. Further supporting the hypothesis that viral DNA transcription contributes to an innate immune response in IIV-6 infected cells, UV-inactivation of IIV-6 significantly reduced the levels of secreted IFN-β in infected MEFs. Similarly, Minamitani et al. showed that UV-inactivation of adenovirus DNA transcription reduced IFN-β induction [47]. Our results also showed that Dicer contributed to IFN-β secretion in response to IIV-6 infection, but further studies would be needed to determine how the role of Dicer in this pathway leads to an innate immune response. Our model highlights a functional homology between the mammalian RLRs and *Drosophila* Dicer-2, since they contribute to the initiation of an immune response to IIV-6 in their respective hosts.

Since IIV-6 is a cytoplasmic DNA virus, it was surprising that induction of IFN-β in primary MEFs was independent of STING. Due to the AT-rich nature of the IIV-6 genome, it may be that, like EBV, IIV-6 encodes genes that are preferentially transcribed by RNA Pol III and whose transcripts are subsequently sensed by RLRs [30,73]. Recently, Lau et al. showed that the presence of viral oncogenes in transformed cell lines blocked STING activity. Specifically, human papilloma virus E7 and adenovirus E1A directly bind to STING through their LXCXE motifs [74]. Recent studies have shown that hepatitis B virus, a DNA virus, stimulates the innate immune response via an NFκB- and RIG-I-dependent, but a cGAS/STING-independent, manner [75–76]. Other large DNA viruses, such as Kaposi’s sarcoma-associated herpesvirus, encode viral proteins, namely vIRF1 and ORF52, that directly inhibit the cGAS/STING signaling axis [7,77]. It is plausible that IIV-6 encodes a gene that also inhibits the cGAS/STING pathway. If IIV-6 does contain such a gene, it would likely have additional functions other than inhibiting mammalian cGAS since insects do not contain a cGAS homolog [78].

The mammalian RLRs are unique proteins in that they contain both CARD domains and an RNA helicase domain. This RNA helicase domain is most closely related to the RNA helicase domain in Dicer [37,79]. Dicer is found in a variety of organisms, including chordates, invertebrates, and plants, and its role in RNAi has provided diverse phyla with an innate immune response to virus infection. Specifically, Dicer-2 in *Drosophila* senses viral RNA to initiate an antiviral response, similar in function to the mammalian RLRs [37,80]. As RLRs are not found in *Drosophila*, it seems plausible that Dicer represents an ancient antiviral mechanism that gave rise to RLRs later in evolution, alongside the IFN system. Mammals likely shifted away from RNAi not because of lack of functionality, but as a mechanism to respond to a broader variety of viral pathogens through the use of IFN [81]. The utility and effectiveness of IFN in mammals may have subsequently rendered mammalian Dicer redundant [82].

As such, it is controversial if mammalian Dicer has a function in innate immunity. Dicer is involved in the antiviral immune response to encephalomyocarditis virus and nodamura virus in mouse embryonic stem cells, hamster fibroblasts, and suckling mice [83–85], and RNAi is a functional antiviral mechanism against influenza A virus *in vitro* and *in vivo* [81]. Conversely, there is no increase in siRNA or viral miRNA during infection of Huh7 cells with DENV or WNV, and there is no change in replication of several arboviruses in HEK 293T cells lacking Dicer compared to wild type [86]. This suggests that mammalian Dicer is not involved in restricting viral infection and replication. Taken together, it is possible that the Dicer-dependent immune response in mammals is contingent on the type of viral infection. Therefore, why IIV-6 infection in Dicer^-/-^ MEFs resulted in reduced IFN-β secretion warrants further investigation.

Our results demonstrating that IIV-6 infection primes the mammalian innate immune response to reduce subsequent arbovirus infection suggest that similar IIV-6-based strategies could be used to protect against WNV and DENV infections. Currently, *Wolbachia*, a Gram-negative bacterium and arthropod endosymbiont are being considered as a mechanism to restrict DENV infection. DENV replicates less in mosquitoes infected with *Wolbachia* [87–88]. Since *Wolbachia* does not infect mammals, the bacteria could be added to the mosquito population to restrict overall DENV replication by producing reactive oxygen species that induce a global immune response [89]. Along these lines, IIV-6 could be used as a non-replicative virus to induce an immune response that can restrict arbovirus infection in mammals. Our results indicating reduced VSV and KUNV replication in the presence of IIV-6 identify an avenue of future research for vaccine or therapeutic development, possibly to identify the specific viral gene that activates a protective mammalian immune response. Before the putative therapeutic value of IIV-6 can be determined, future research should be performed to determine if IIV-6 can be delivered in insect saliva, with or without an arbovirus present. Studies could also be performed to ask if an IIV-6-mediated protective immune response could be induced in an insect host, such as the mosquito, to potentially reduce arboviral load in the animals.

In conclusion, we demonstrate a novel example of an insect DNA virus eliciting a mammalian immune response via RNA Pol III and the RLR pathway. While it is unknown if the IIV-6-mediated immune response contributes to the restriction of its own replication in mammalian cells through an unidentified mechanism, we show that the mammalian immune response to IIV-6 reduces infection by arboviruses VSV and KUNV, which have implications for the development of therapeutics to viruses such as WNV.

## Supporting Information

S1 Fig Inactivated IIV-6 does not elicit an immune response in MEFs. MEFs were infected with IIV-6, (A) heat-inactivated IIV-6, or (B) UV-inactivated IIV-6 at an MOI of 1 TCID_50_/cell. Supernatant from infected cells was collected and analyzed for IFN-β secretion with ELISA. Each virus infection was compared to mock for each time point, and all experiments were completed in biological duplicate (*, *P* < 0.05).
(TIF)

S2 Fig IIV-6 causes a cytopathic effect in MEFs. (A) MEFs were infected with IIV-6 at an MOI of 1 TCID_50_/cell and imaged at 24 hours post-infection by phase contrast microscopy. Infected cells have a rounded morphology, unlike the mock-infected MEFs. (B) Mock- or IIV-6-infected cells were stained with trypan blue and counted using a hemocytometer. Each bar represents two groups of 300 counted cells. (C) Mock- or IIV-6-infected cells were labeled with propidium iodide for cell death and counted using flow cytometry. Two groups of 10,000 cells were counted for each condition (*, *P* < 0.05; **, *P* < 0.001).
(TIF)

S3 Fig IIV-6 does not replicate in MEFs. (A) MEFs or (B) S2 cells were infected with IIV-6 at an MOI of 1 TCID_50_/cell. 24 hours post-infection cells were collected and genomic DNA was isolated and quantified for IIV-6 capsid levels by qPCR. (B) The number of genome copies increased significantly in S2 cells from 0 to 48 hours post-infection and beyond (*, *P* < 0.01). (C-D) MEFs were infected with IIV-6 at an MOI of 1 TCID_50_/cell, supernatant and cells were collected, and intracellular virions were obtained from cells using a freeze-thaw method. (C) Cell culture supernatant and (D) intracellular virus from infected MEFs were titered onto insect S2 cells to determine viral load over time. Gray boxes indicate the range of S2 cell death from the dilution of MEF infection media. No significant difference in viral titer over time was observed. All assays were completed in biological duplicate.
(TIF)

S4 Fig IFN-β induction following IIV-6 infection is reduced in the absence of RIG-I. STING^-/-^, RIG-I^-/-^, MDA5^-/-^, Dicer^-/-^, or MAVS^-/-^ MEFs and their corresponding wild-type MEFs were infected with IIV-6 at an MOI of 1 TCID_50_/cell in biological duplicate. Total RNA was collected at 24 hours post-infection and analyzed for IFN-β mRNA using qRT-PCR. Knockout cell lines were compared to their wild-type counterparts (*, *P* < 0.05).
(TIF)

S5 Fig IIV-6 does not replicate in RIG-I-deficient MEFs. RIG-I^+/+^ and RIG-I^-/-^ MEFs were infected with IIV-6 at an MOI of 1 TCID_50_/cell. Supernatants were collected and titered onto S2 cells to determine viral load over time. IIV-6 concentration did not increase significantly by 5 days post-infection in MEFs.
(TIF)

S6 Fig IIV-6 removal and cell viability during priming and co-infection experiments. (A) Supernatant from mock- or IIV-6-infected MEFs was collected, centrifuged at 15,000 RCF for 10 minutes [44] and titered onto S2 cells to determine viral load. Supernatant from IIV-6-infected cells contains no detectable levels of virus after centrifugation. Assay was completed in biological and technical triplicate. MEF cell viability from (B) IIV-6 priming experiments and (C) co-infection experiments were quantified with trypan blue staining. Four groups of cells were counted for each sample group, and statistical analysis was performed to compare mock priming or co-infection to the IIV-6 counterpart (*, *P* < 0.05). Representative brightfield images indicate increased cell death during infection.
(TIF)
