# Supplementary figures and images for: Invertebrate Iridescent Virus 6, a DNA Virus, Stimulates a Mammalian Innate Immune Response through RIG-I-Like Receptors
Source: PLoS One. 2016 Nov 8;11(11):e0166088. doi: 10.1371/journal.pone.0166088 (PMC5100955; doi:10.1371/journal.pone.0166088)

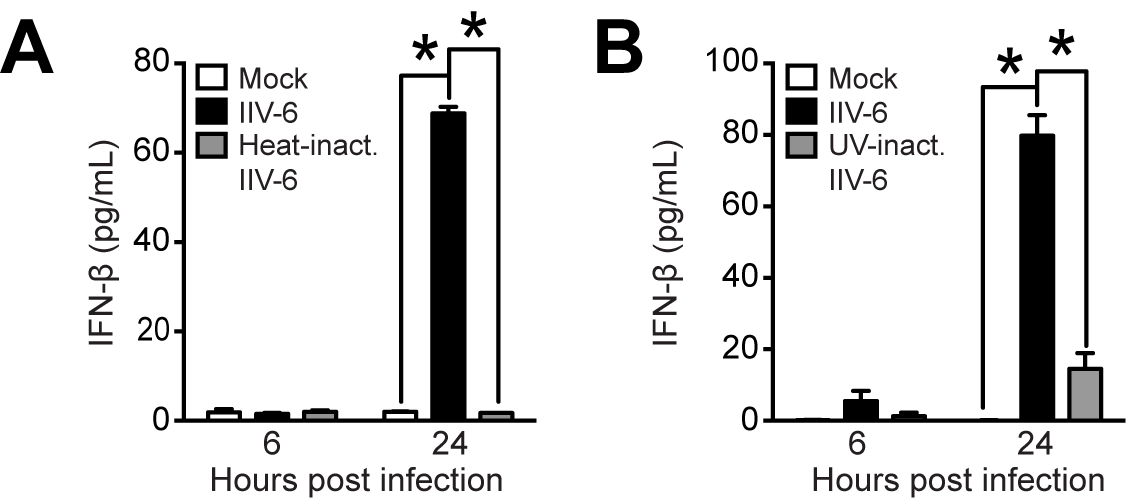

Supplement: S1 Fig — MEFs were infected with IIV-6, (A) heat-inactivated IIV-6, or (B) UV-inactivated IIV-6 at an MOI of 1 TCID50/cell. Supernatant from infected cells was collected and analyzed for IFN-β secretion with ELISA. Each virus infection was compared to mock for each time point, and all experiments were completed in biological duplicate (*, P < 0.05). (TIF) [file pone.0166088.s001.tif]

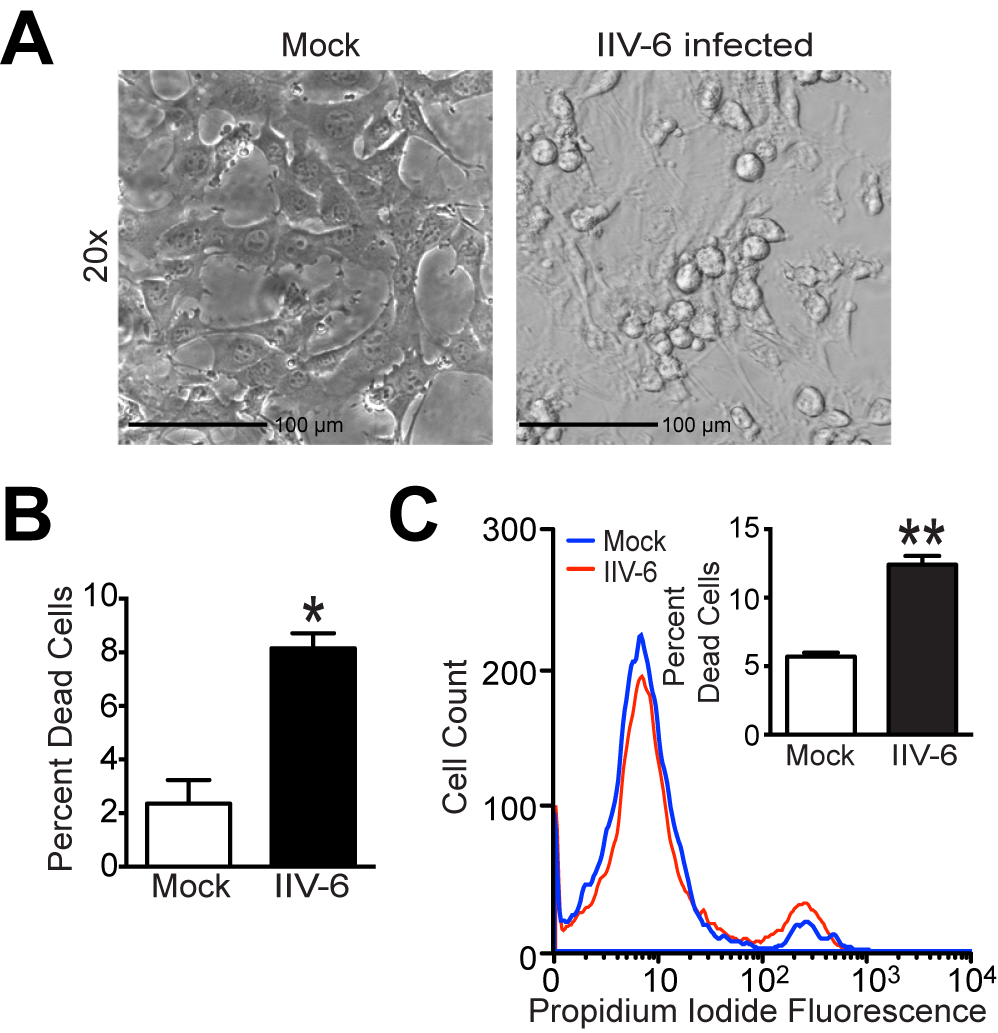

Supplement: S2 Fig — (A) MEFs were infected with IIV-6 at an MOI of 1 TCID50/cell and imaged at 24 hours post-infection by phase contrast microscopy. Infected cells have a rounded morphology, unlike the mock-infected MEFs. (B) Mock- or IIV-6-infected cells were stained with trypan blue and counted using a hemocytometer. Each bar represents two groups of 300 counted cells. (C) Mock- or IIV-6-infected cells were labeled with propidium iodide for cell death and counted using flow cytometry. Two groups of 10,000 cells were counted for each condition (*, P < 0.05; **, P < 0.001). (TIF) [file pone.0166088.s002.tif]

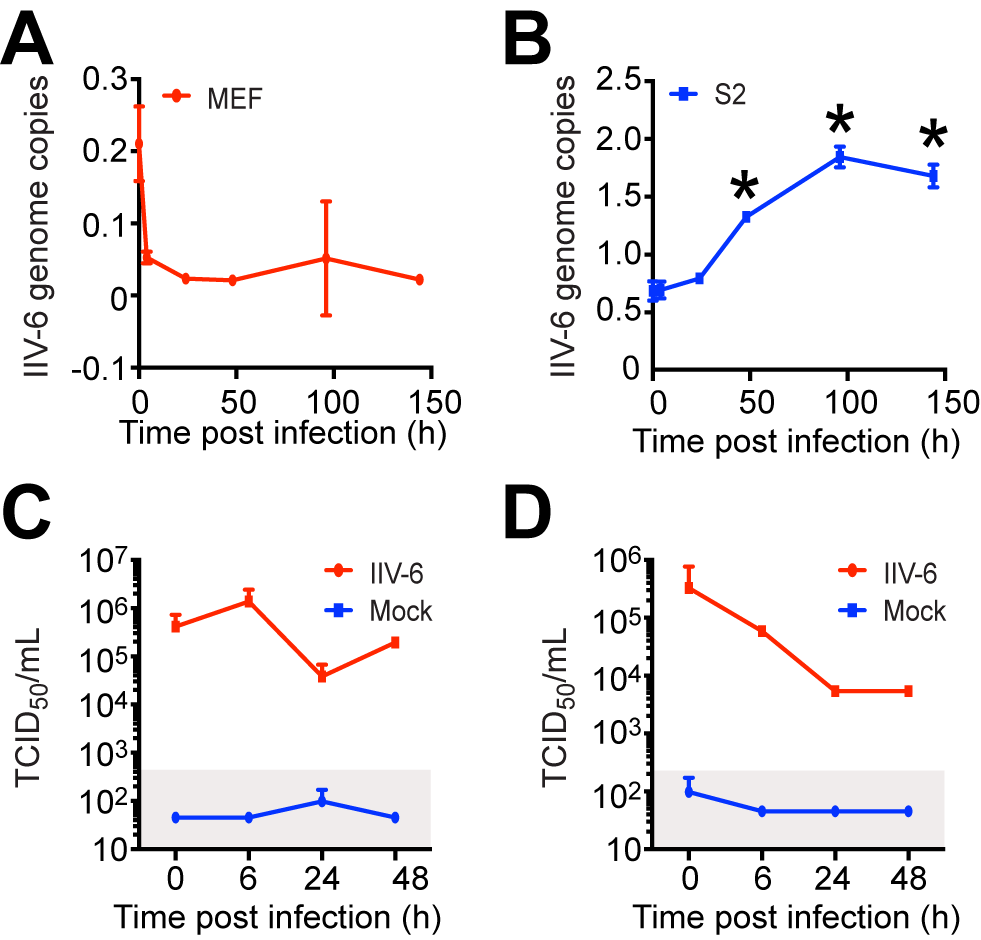

Supplement: S3 Fig — (A) MEFs or (B) S2 cells were infected with IIV-6 at an MOI of 1 TCID50/cell. 24 hours post-infection cells were collected and genomic DNA was isolated and quantified for IIV-6 capsid levels by qPCR. (B) The number of genome copies increased significantly in S2 cells from 0 to 48 hours post-infection and beyond (*, P < 0.01). (C-D) MEFs were infected with IIV-6 at an MOI of 1 TCID50/cell, supernatant and cells were collected, and intracellular virions were obtained from cells using a freeze-thaw method. (C) Cell culture supernatant and (D) intracellular virus from infected MEFs were titered onto insect S2 cells to determine viral load over time. Gray boxes indicate the range of S2 cell death from the dilution of MEF infection media. No significant difference in viral titer over time was observed. All assays were completed in biological duplicate. (TIF) [file pone.0166088.s003.tif]

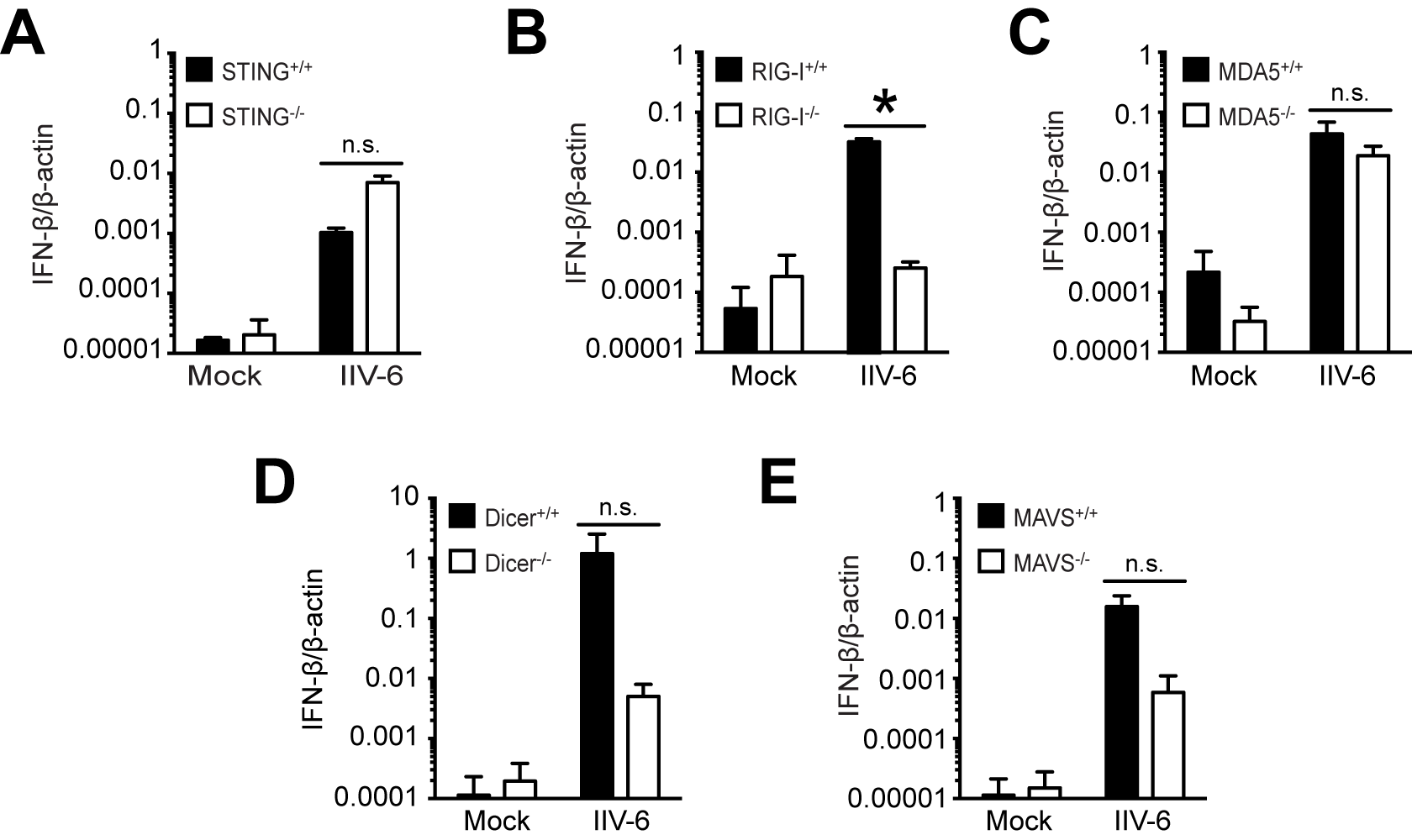

Supplement: S4 Fig — STING-/-, RIG-I-/-, MDA5-/-, Dicer-/-, or MAVS-/- MEFs and their corresponding wild-type MEFs were infected with IIV-6 at an MOI of 1 TCID50/cell in biological duplicate. Total RNA was collected at 24 hours post-infection and analyzed for IFN-β mRNA using qRT-PCR. Knockout cell lines were compared to their wild-type counterparts (*, P < 0.05). (TIF) [file pone.0166088.s004.tif]

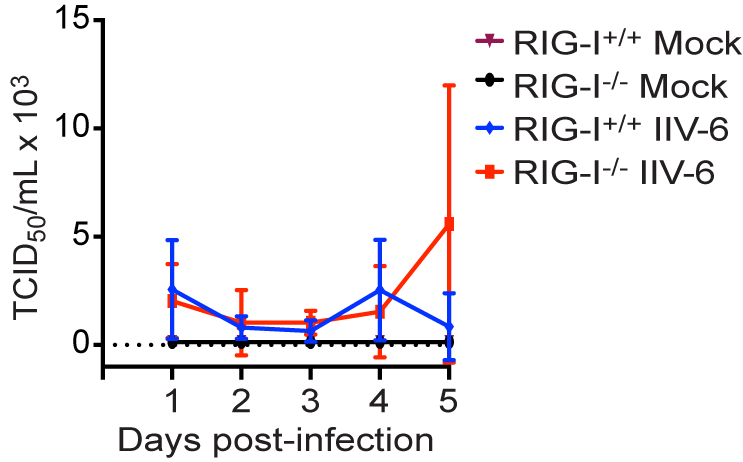

Supplement: S5 Fig — RIG-I+/+ and RIG-I-/- MEFs were infected with IIV-6 at an MOI of 1 TCID50/cell. Supernatants were collected and titered onto S2 cells to determine viral load over time. IIV-6 concentration did not increase significantly by 5 days post-infection in MEFs. (TIF) [file pone.0166088.s005.tif]

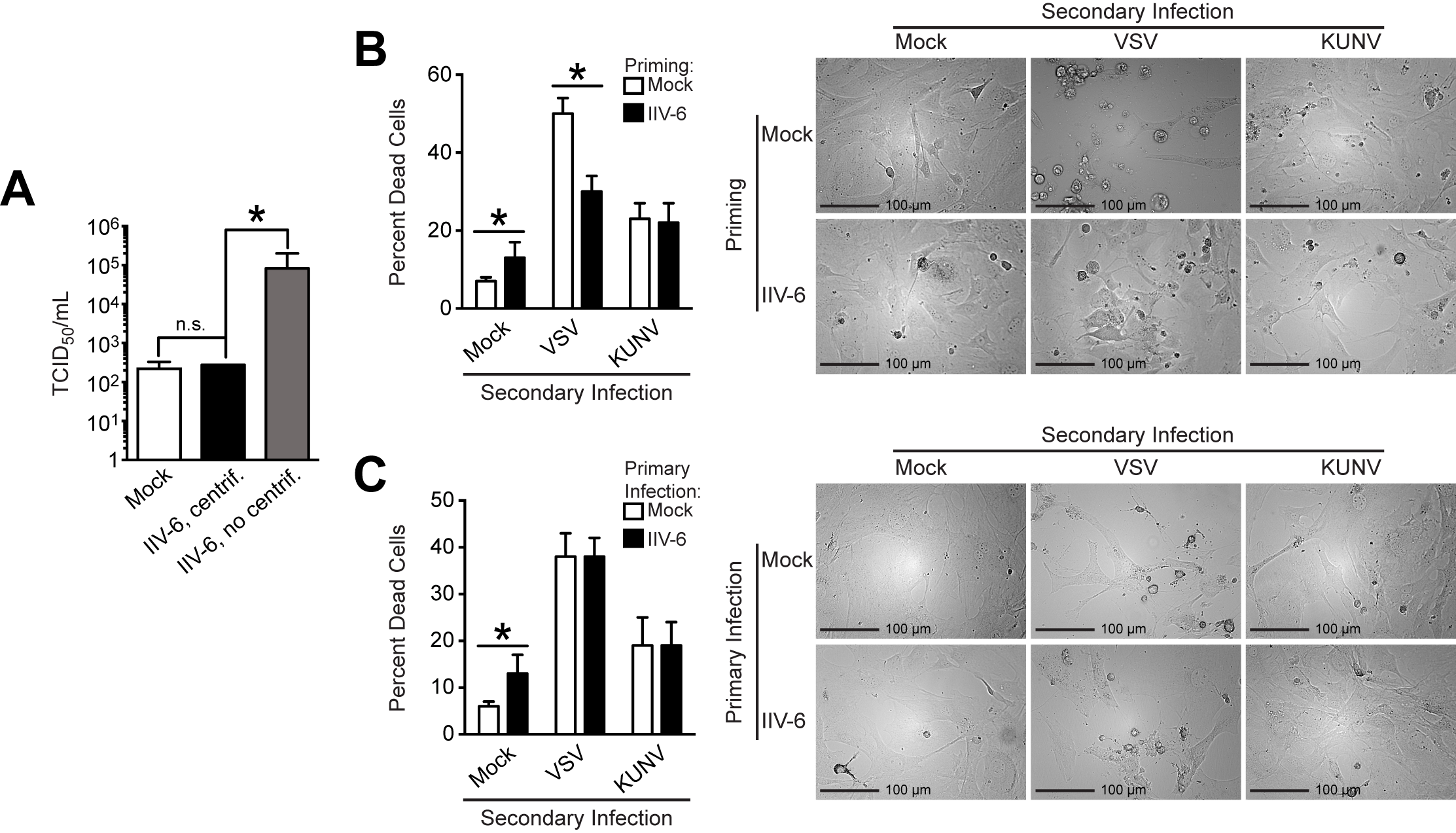

Supplement: S6 Fig — (A) Supernatant from mock- or IIV-6-infected MEFs was collected, centrifuged at 15,000 RCF for 10 minutes [44] and titered onto S2 cells to determine viral load. Supernatant from IIV-6-infected cells contains no detectable levels of virus after centrifugation. Assay was completed in biological and technical triplicate. MEF cell viability from (B) IIV-6 priming experiments and (C) co-infection experiments were quantified with trypan blue staining. Four groups of cells were counted for each sample group, and statistical analysis was performed to compare mock priming or co-infection to the IIV-6 counterpart (*, P < 0.05). Representative brightfield images indicate increased cell death during infection. (TIF) [file pone.0166088.s006.tif]
